# Adjuvant Pam3CSk4 does not improve the immunization against *Cryptococcus gattii* infection in C57BL/6 mice

**DOI:** 10.7717/peerj.14778

**Published:** 2023-01-31

**Authors:** Gabriela Yamazaki de Campos, Patrícia Kellen Martins Oliveira-Brito, Júlia Garcia Guimarães, Letícia Serafim da Costa, Javier Emílio Lazo Chica, Thiago Aparecido da Silva

**Affiliations:** 1 Department of Cell and Molecular Biology and Pathogenic Bioagents, University of Sao Paulo, Ribeirao Preto, Sao Paulo, Brazil; 2 Department of Microbiology, Institute of Biomedical Sciences, University of Sao Paulo, Sao Paulo, Brazil; 3 Institute of Natural and Biological Sciences, Federal University of Triângulo Mineiro, Uberaba, Minas Gerais, Brazil

**Keywords:** *Cryptococcus gattii*, TLR2, Vaccine, Pam3CSK4, Immunomodulation

## Abstract

**Background:**

Cryptococcosis is a relevant invasive fungal infection that affects immunocompromised and immunocompetent individuals when caused by *Cryptococcus gattii*. Host innate and adaptive immune responses can be subverted by *C. gattii,* that blocks the differentiation of T helper (Th) 1 and Th17 cells, which are involved in the protection against cryptococcosis. Moreover, the macrophage polarization is modulated by *C. gattii* infection that requires a balance in the macrophage subsets to control the *C. gattii* infection. Toll-like receptor (TLR) 2 agonists are important immunomodulators favoring a pro-inflammatory response with potential fungicidal activity, and TLR2 agonists have been used as adjuvants in vaccines against infections caused by bacteria or viruses. Therefore, this work aimed to evaluate the immunomodulatory effect of the tripalmitoyl lipopeptide S-glycerol cysteine (Pam3CSK4 or P3C4), a TLR2 agonist, as an adjuvant in the vaccination against *C. gattii* infection.

**Methods and Results:**

C57BL/6 mice were immunized with 2 × 10^7^ inactivated yeasts of *C. gattii* via intranasal route on day 1, 14 and 28 (Immunized group). Immunization was associated with 1µg or 10µg of adjuvant P3C4 (Immunized+P3C4-1µg or Immunized+P3C4-10 µg), followed by *C. gattii* infection on day 42 after the immunization protocol. Immunized+P3C4-1 µg group had reduced levels of IgG1, IgG2a and IgA and no significant difference in the IgG and IgM anti-GXM antibody titer was detected, compared to the Immunized group. High levels of IL-17 and IL-1*β* in lung tissue of mice from the Immunized+P3C4-1µg group did not promote a predominance of Th17 cells, in contrast, the frequency of TLR2^+^ cells was increased in immunized mice that received 1 µg of P3C4. The reduction in the relative expression of T-bet and high levels of Foxp3 detected in the lungs of the Immunized+P3C4-1µg group suggest a prevalence of regulatory T cells in the tissue, which did not contribute to the control of *C. gattii* infection. The immunization protocol associated with 10 µg of adjuvant P3C4 induced high levels of IL-17 in the lung tissue, whereas the levels of pro-inflammatory cytokines were downregulated. To evaluate the effect of adjuvant P3C4 in the control of *C. gattii* infection, quantification of the fungal burden in the lungs was performed by the CFU assay, and the groups with adjuvant P3C4 showed a pulmonary *C. gattii* burden that was not significantly altered when compared with the immunized group. The mice that received 1 µg of adjuvant P3C4 had a lower percentage of inflammatory infiltrate in the lungs.

**Conclusion:**

The immunomodulatory effect of P3C4, associated with the immunization protocol, plays an imbalance between pro- and anti-inflammatory response in the lungs that did not favor a protection against *C. gattii* infection, which is related to the immune response characterized by a suppressive/regulatory profile in the pulmonary microenvironment after *C. gattii* infection.

## Introduction

*Cryptococcus gattii* is a relevant fungal species that causes disseminated infection, namely cryptococcosis, in immunocompromised patients as well as in healthy individuals (O’Halloran, Powderly & Spec, 2017; Park et al., 2009). The most susceptible patients are those who require prolonged use of antibiotics or immunosuppressive therapies, chemotherapy, or patients with acquired immunodeficiency syndrome (AIDS). Human infection caused by *C. gattii* is initiated *via* inhalation of dehydrated yeasts that are deposited in the pulmonary alveoli. In the first stage of infection, innate immunity is crucial for the initial recognition of yeasts by alveolar macrophages, dendritic cells, and epithelial cells. Macrophages have plasticity that alters their phenotype according to the microenvironment, and two major subsets are characterized: classical M1 activated and alternative M2 activated cells (Mantovani et al., 2004; Shapouri-Moghaddam et al., 2018). The M1 subtype has inducible nitric oxide synthase (iNOS) as its main molecular marker and produces high levels of pro-inflammatory cytokines and reactive oxygen species (ROS) (Davis et al., 2013). The M2 subtype has Arginase-1 as the main marker and is involved in tissue repair and regulatory roles (Gordon & Martinez, 2010). Dendritic cells are important antigen presenters for naive T cells, leading to the differentiation of this cell type into Th1, Th2, or Th17 subsets that can be characterized by their major transcription factors, such as T-bet, STAT1, and STAT4 (Th1); GATA3 and STAT6 (Th2); and ROR *γ*T and STAT3 (Th17) (Zhu, Yamane & Paul, 2010).

Protection against cryptococcosis is typically associated with the pro-inflammatory response induced by Th1 cells with the production of IFN-*γ*, interleukin (IL)-2, and IL-12, unlike the Th2 subset, which is associated with an anti-inflammatory response (Campuzano & Wormley, 2018). However, studies have demonstrated that *Cryptococcus spp.* can still escape the immune response of the host, attenuating the role of lung dendritic cells since their maturation is compromised by blocking the release of TNF-*α* and chemokines (Angkasekwinai et al., 2014; Huston et al., 2013). *Cryptococcus* spp. can survive and replicate inside phagolysosomes, leading to their release into the extracellular environment through lytic exocytosis or vomocytosis. Furthermore, *Cryptococcus* spp. can adapt to stress in the lung environment through the expansion of its cell size and capsule thickness, compromising the phagocytic and fungicidal activity of the immune cells, and smaller-sized yeasts can be generated, contributing to the systemic spread of the pathogen (Gerstein et al., 2015; O’Meara, Andrew & Alspaugh, 2012; Perfect & Casadevall, 2002; Ruma-Haynes, Brownlee & Sorrell, 2000). *Cryptococcus* spp. developed several mechanisms to subvert the host immune response, and the polysaccharide capsule of yeast is considered the major virulence factor involved in the immunosuppression and inhibition of the recognition of yeasts by innate immune cells (Elsegeiny, Kieren & Williamson, 2018; Shourian & Qureshi, 2019). Glucuronoxylomannan (GXM) represents more than 90% of the capsule, and GXM’s structure is composed of the mannosyl residues that can be acetylated or replaced by other sugar moieties, then GXM’s structure varies between *Cryptococcus* species and also is highly influenced by the microenvironment (Cherniak & Sundstrom, 1994; Urai et al., 2016).

Toll-like receptors (TLRs) are a family of pattern recognition receptors (PRRs) that are composed of 12 receptors in mice. These receptors are produced in the endoplasmic reticulum and recruited to the cell membrane or intracellular compartments, such as endosomes (Botos, Segal & Davies, 2011; Kawasaki & Kawai, 2014). After the recognition of pathogen-associated molecular patterns (PAMPs), TLRs dimerize and recruit adaptor proteins such as MYD88 and TRIF, which leads to the activation of several transcription factors and protein kinases, such as NK- *κ*B, IRFs, and/or MAP kinase (Pålsson-McDermott & O’Neill, 2007). TLR2 forms dimers with TLR1 or TLR6 on the cell membrane and recognizes several microbial components such as peptidoglycans from gram-positive bacteria (Aderem & Ulevitch, 2000), zymosan from fungi (Sato et al., 2003), phospholipomannan from *Candida albicans* (Jouault et al., 2003), and glycolipids from *Treponema maltophilum* (Opitz et al., 2001). Synthetic ligands are widely used in TLR2 immunomodulation assays, such as dipalmitoyl lipopeptide S-glycerol cysteine (Pam2CSK4) and tripalmitoyl lipopeptide S-glycerol cysteine (Pam3CSK4 or P3C4) (Brandt et al., 2013; Shibata et al., 2000). Cell activation *via* TLR2 contributes to the control of phagosome maturation, allowing better association of the antigen with major histocompatibility complex II (MHC-II), which leads to increased expression of this complex on the cell surface. Furthermore, dendritic cell (DC) presentation of epitopes *via* MHC-II to specific T cells increases in the presence of the TLR2 agonist P3C4 (Khan et al., 2007). Therefore, some immunization protocols use the covalent association of antigens and agonists to enhance cellular and humoral immune responses (Cote-Sierra et al., 2002; Khan et al., 2007; Zeng et al., 2002).

Vaccines with attenuated or inactivated fungal agents are relevant candidates for immunomodulation and long-term immune responses; therefore, they can be effective in immunocompetent patients. The use of adjuvants, such as complete Freund’s solution (CFA), associated with the culture filtrate antigen (CneF) of *Cryptococcus neoformans* could partially protect against *C. neoformans* infection (Murphy et al., 1998). There was a significant reduction in lung fungal burden in animals vaccinated with cell wall and capsule proteins of *C. gattii* associated with the adoptive transfer of dendritic cells (DC) pulsed with an acapsular strain of *C. gattii* (ΔCAP60) (Chaturvedi et al., 2014; Specht et al., 2015; Ueno et al., 2015). The production of TNF-*α*, IFN-*γ*, and IL-17 is essential for these strategies to provide protection against infection by *C. gattii* (Chaturvedi et al., 2014; Specht et al., 2015; Ueno et al., 2015). In addition, our group recently demonstrated that administration of a Dectin-1 agonist as an adjuvant in an immunization strategy utilizing C57BL/6 mice can also provide high levels of IFN- *γ* and IL-10, decreasing the burden of *C. gattii* in the lungs (Oliveira-Brito et al., 2022). To date, no studies have evaluated the use of the agonist P3C4 to combat fungal infections; however, several studies have demonstrated its effects in other infectious diseases. P3C4 can induce a Th1 response through the activation of antigen-presenting cells (APCs), leading to increased survival in mice. This was demonstrated in a vaccine strategy against the hepatitis C virus (Chua et al., 2008). The TLR2 activation pathway *via* MYD88 signaling favors the production of antibodies in a T cell-dependent manner, contributing to the longevity of antibody-producing B cells (Komegae et al., 2013).

The development of vaccines to combat cryptococcosis is an advancement aimed at mitigating drug-resistant organisms and antifungal side effects. The present study evaluated the immunomodulatory effects of P3C4, a TLR2 agonist, as an adjuvant in an immunization strategy against *C. gattii*. Doses of 1 µg and 10 µg of adjuvant P3C4 were tested, and it was demonstrated that 10 µg of P3C4 reduced the levels of pro-inflammatory cytokines in the lung tissue and did not alter the subtypes of CD4^+^ T cells. The administration of 1 µg of P3C4 induced the predominance of Th17-associated cytokines in the lung tissue and a prevalence of Foxp3 transcription factor that characterizes regulatory CD4^+^ T cells (Treg). However, this microenvironment in the lung tissue is not sufficient to control *C. gattii* infection.

## Materials & Methods

### Animals

Male C57BL/6 mice (6–8 weeks old) were acquired from the animal facility at the Campus of Ribeirão Preto of the University of São Paulo, Brazil. The mice were housed in the animal facility of the Department of Cell and Molecular Biology and Pathogenic Bioagents of Ribeirão Preto Medical School, and all animal experiments were performed according to the Ethical Principles Guide for the Care and Use of Laboratory Animals adopted by the Brazilian College of Animal Experimentation. The protocols were approved by the Committee on Ethics in Animal Research of Ribeirão Preto Medical School (protocol 072/2019). Mice were anesthetized with ketamine hydrochloride (20.0 mg/kg, i.p.) and xylazine hydrochloride (2 mg/kg, i.p.) as previously reported (Oliveira-Brito et al., 2022), and the euthanasia was performed in the end period of *C. gattii* infection.

### Culture of *Cryptococcus gattii* and heat inactivation of yeasts

R265 *C. gattii* strain (B serotype, VGII molecular genotype) was recovered from 25% glycerol stocks stored at −80 °C and cultured on Sabouraud dextrose medium at 30 °C with constant agitation (150 rpm) for 16–18 h. *C. gattii* yeast with a thin capsule (*C. gattii*-thin) were obtained after inoculation in YPD medium containing 0.5 M NaCl, as described by Ikeda-Dantsuji et al. (2015), and incubated at 30 °C with constant agitation at 150 rpm for 20–24 h. Yeast cells were harvested by centrifugation at 7,600 × g for 10 min at 25 °C and washed three times with sterile PBS (Thermo Fisher Scientific, Waltham, MA, USA). *C. gattii*-thin was heat-inactivated at 70 °C for 1 h (HK-*C. gattii*-thin). The cell concentration was determined using a Neubauer chamber with Chinese ink. A fraction of the HK-*C. gattii*-thin yeast was spread on Sabouraud dextrose agar to confirm the heat inactivation.

C57BL/6 mice were infected with viable yeast of *C. gattii* that were cultured on Sabouraud dextrose medium at 30 °C with constant agitation (150 rpm) for 16–18 h. Yeast cells were harvested by centrifugation at 7,600 × g for 10 min at 25 °C and washed three times with sterile PBS (Thermo Fisher Scientific). Cell concentration was determined using a Neubauer chamber with Chinese ink.

### Protocol of immunization of mice in association with adjuvant P3C4

Mice were anesthetized with ketamine hydrochloride (20.0 mg/kg, i.p.) and xylazine hydrochloride (2 mg/kg, i.p.). Animals were immunized with a cell suspension of 2 × 10^7^ H.K.-*C. gattii* with or without P3C4 (1 or 10 µg/mouse) intranasally, and the control group received PBS alone (*n* = 5 in each group). Immunization was performed three times with a two-week interval among them (on day 1, 14 and 28). The animals were separated into the following groups: PBS, Immunized, Immunized +P3C4 (1 µg), and Immunized +P3C4 (10 µg). On day 35, the blood was collected to measure the levels of IgM and IgG anti-GXM. On day 42, mice were anesthetized and infected with 1 × 10^5^ *C. gattii* yeast cells intranasally. Fourteen days after infection (day 56), the animals were euthanized to collect blood samples and lungs.

### Fungal burden in the lungs measured by CFU

The lungs of the animals were removed 14 days after *C. gattii* infection (day 56), and the tissue was stored in one mL cold 1x sterile PBS buffer (pH 7.2) and immediately weighed and homogenized (IKA Werke, Staufen, Germany). Suspensions of homogenized lung tissue were diluted and plated on Sabouraud dextrose (Oxoid) agar medium containing 100 µg/mL ampicillin. The plates were incubated at 30 °C for 48 h, and the *C. gattii* colonies were counted to perform the colony forming unit (CFU) assay, which is expressed in CFU/g.

### Serum immunoglobulin isotyping

After 14 days of *C. gattii* infection (day 56), the serum was obtained from all mice to quantify the levels of the immunoglobulins isotyping (IgG1, IgG2a, IgG2b, IgG3, IgM, and IgA along with kappa and lambda light chains) using the Easy-Titer™ Mouse IgG Assay Kit (Thermo Fischer Scientific) as previously reported (Oliveira-Brito et al., 2022).

### Quantification of the levels of IgG and IgM antibodies specific to GXM

The levels of IgG and IgM anti-GXM in the serum of the animals were measured 7 days after the immunization protocol (day 35) and it was performed in 96-well microplates coated with 20 µg/mL GXM isolated from *C. gattii*, as described by Wozniak and Levitz (Wozniak & Levitz, 2009), and incubated overnight at 4 °C. The plate was washed three times with wash buffer (PBS-Tween 20; 0.05%) and incubated with MOLICO^®^ skim dry milk powder (1.0%) for 2 h at 25 °C (room temperature, RT) for blocking. The plate was washed five times and mouse serum was added and incubated for 2 h at RT, furthermore, the plate was washed five times. Then, goat anti-mouse IgM (*μ*-chain specific)-peroxidase antibody (1:2500, Cat# A8786; Sigma-Aldrich, St. Louis, MO, USA) or rabbit anti-mouse IgG (whole molecule)–peroxidase antibody (1:5000, Cat# A9044, Sigma-Aldrich) was added in the plate and incubated for 1 h. The plates were washed seven times and 50 µL 3,3′,5,5′-tetramethylbenzidine (TMB) substrate solution was added. After 30 min of incubation at RT, 30 µL of stop solution (2N H_2_SO_4_) was added. Absorbance was recorded at 450 nm using a spectrophotometer (Power Wave X; BioTek Instruments, Winooski, VT, USA).

### Quantification of cytokine levels in the lungs

Lung homogenates of the animals were obtained 14 days after *C. gattii* infection (day 56). The tissue was stored in one mL cold 1x sterile PBS buffer (pH 7.2), immediately weighed, and homogenized (IKA Werke, Staufen, Germany). The supernatant was collected after centrifugation (3,220 × g, 10 min) and a protease inhibitor was added (phenylmethylsulfonyl fluoride, PMSF). Cytokine levels were quantified by ELISA using the OptEIA™ Kit (Pharmingen, San Diego, CA, USA) according to the manufacturer’s instructions. IFN-*γ*, IL-12(p70), IL-10, IL-4, IL-17A, TNF-*α*, IL-1*β*, IL-2, and IL-6 levels were measured in the lungs, and IFN-*γ*, IL-17, and IL-4 levels were quantified in the serum. The levels of cytokines in the tissues were normalized relative to the organ mass (mg) and expressed in pg/mL.

### Phenotyping of immune cells in the pulmonary tissue by flow cytometry

Pulmonary leukocytes were obtained as previously described by Oliveira-Brito et al. (2020). Prior to phenotyping the cell populations in the lungs, the concentrations of the cell suspensions were determined using a Neubauer chamber, and 1 × 10^6^ cells/mL from each sample were stained with antibodies conjugated to fluorophores. Cells were incubated for 30 min at 4 °C with 0.5 µg of anti-CD16/CD32 monoclonal antibody (Fc block, clone 2.4G2; BD Pharmingen, San Diego, CA). Subsequently, 5 µg/mL of the following antibodies (obtained from BD Pharmingen) were used: anti-CD3 (PE-Cy5 Rat anti-Mouse CD3; clone 17A2), anti-F4/80 (FITC rat anti-mouse F4/80; clone BM8), anti-CD11c (PE Hamster Anti-Mouse CD11c; clone HL3) and anti-TLR2-PE (PE rat anti-mouse TLR2; clone 6C2). The cells were incubated for 45 min with the antibodies, and washed twice with PBS buffer and fixed with PBS-formaldehyde (1%). After these steps, the percentage of cells positive for CD3, F4/80, or TLR2 was measured using flow cytometry (Guava easyCyteTM Mini System).

### Analysis of the profile of CD4^+^ T cells and macrophage subtypes in the lung tissue by qRT-PCR

Total RNA was isolated from the lung cells using TRIzol reagent (Life Technologies, Carlsbad, CA, USA) according to the manufacturer’s instructions. Total RNA was converted to cDNA using an iScript cDNA Synthesis Kit (Bio-Rad, CA, USA). qRT-PCR was performed in a final volume of 10 µL using the EvaGreen Supermix (Bio-Rad). The CFX96 Touch System (Bio-Rad, Hercules, CA, USA) was used under the following reaction conditions: 95 °C for 30 s, 40 cycles of 95 °C for 5 s, and 91.5 °C -−95 °C for 5 s. CD4 ^+^ T cell differentiation was determined by the relative expression of T-bet, ROR *γ*t, GATA-3, Foxp3, and TGF-*β* transcripts, and macrophage polarization was evaluated by the relative transcript expression of Arginase-1, iNOS, and IL-23. Relative transcript expression was quantified using the ΔΔCt method and normalized to *β*-actin expression. The primers used for PCR reaction were: *β*-actin (F: 5′-CCTAAGGCCAACCGTGAAAA-3′/R: 5′-GAGGCATACAGGGACAGCACA-3′), T-bet (F: ′-CACTAAGCAAGGACGGCGAA-3′/R: 5′-CCACCAAGACCACATCCAC-3′), ROR *γ*T (F: 5′-TGGAAGATGTGGACTTCGTT-3′/R: 5′-TGGTTCCCCCAAGTTCAGGAT-3′), GATA-3 (F: 5′-AAGAAAGGCATGAAGGACGC-3′/R: 5′-GTGTGCCCATTTGG ACATCA-3′), Arginase-1 (F: 5′-GTTCCCAGATGTACCAGGATTC-3′/R: 5′-CGATGTCTT TGGCAGATATGC-3′), iNOS2 (F:5′-CCGAAGCAAACATCACATTCA-3′/R: 5′-GGTCTAAAGGCTCCGGGCT-3′), IL-23 (F: 5′TCCGTTCCAAGATCCTTCGA3′/R: 5′TGTTGGCACTAAGGGCT CAG3′), Foxp3 (F: 5′-ATTGAGGGTGGGTGTCAGGA-3′/R: 5′-TCCAAGTCTCGTCTGAAGGCA-3′), and TGF-*β* (F: 5′-GACTCTCCACCT GCAAGACC-3′/R: 5′-GGGACTGGCGAGCCTTAGTT-3′).

### Histopathological and morphometric analysis of the pulmonary tissue

The procedures to obtain the lungs and the preparation of tissue samples for histology were performed as reported by (Oliveira-Brito et al., 2022; Oliveira-Brito et al., 2022). Moreover, the sections were stained with hematoxylin and eosin (H&E), Grocott–Gomori methenamine silver and mucicarmine to be evaluated as previously reported (Oliveira-Brito et al., 2022). The images acquired were used to quantify the percentage of inflammatory infiltrate using a semi-automated quantitative manner using ImageJ Fiji. Quantification of inflammatory infiltrates was performed in four sections (6,3X magnification) subdivided into grids (269568.2 µm^2^) in a total area of one mm^2^ per animal.

### Statistical analysis

All data were analyzed using Prism 7.0 (GraphPad Software, San Diego, CA, USA), and the results were expressed as mean ± standard deviation (SD). Homogeneity of variance was analyzed using the Kolmogorov–Smirnov test. For all data with Gaussian distribution, an ANOVA test followed by Bartlett’s test was applied to the three groups of experiments (PBS, Immunized and Immunized-1 µg, or Immunized-10 µg). In all data with a non-normal distribution, the Kruskal–Wallis test was applied to the three groups of experiments. To determine the differences between the means of groups (*p* <0.05), one-way ANOVA followed by Tukey’s multiple comparisons test or Kruskal–Wallis test followed by Dunn’s multiple comparisons test was performed.

## Results

### Adjuvant P3C4 does not favor the development of immunoglobulin isotypes with effector function against *C. gattii*

Previous studies demonstrate the importance of TLR2 agonists in inducing a pro-inflammatory response that culminates in the control of infectious diseases (Chua et al., 2008; Komegae et al., 2013; Salgado et al., 2019). Intranasal administration of TLR2 agonists can provide protection against several pathogens, including SARS-CoV-2 and influenza viruses (Proud et al., 2021; Tan et al., 2012), and gram-negative bacteria *Chlamydia trachomatis* (Cheng et al., 2011a), and *Bordetella pertussis* (Allen et al., 2018). The immunomodulatory activity of the TLR2 agonist (Pam3CSK4 or P3C4) has not been evaluated as an adjuvant associated with an immunization protocol to combat fungal infections. Therefore, this work performed the immunization of mice *via* intranasal administration, using heat-killed *C. gattii* yeast associated with the adjuvant P3C4 to control *C. gattii* infection.

**Figure 1:**
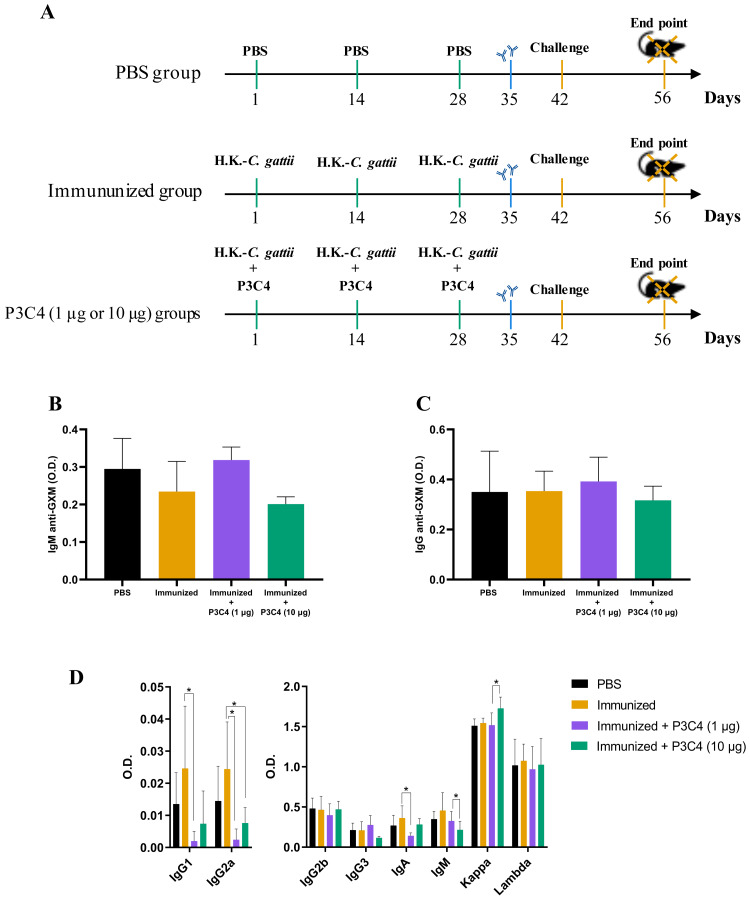
Levels of immunoglobulins in the serum of immunized mice in association with adjuvant P3C4. (A) C57BL/6 mice were immunized on day 1, 14 and 28, with a cell suspension of 2 × 10^7^ H. K.-*C. gattii* with or without Pam3CSk4 (P3C4, 1 µg or 10 µg/mouse) intranasally, while the control group received the vehicle alone (PBS). On day 42 the immunized mice were infected with 1 × 10^5^ *C. gattii* yeast cells, and the mice were euthanized on day 56. On day 35, the levels of (B) immunoglobulin M (IgM) and (C) IgG anti-glucuronoxylomannan (GXM) were measured in the serum by ELISA. (D) On day 56, the serum was obtained to quantify the immunoglobulin isotypes by ELISA. The values are expressed as O.D (450 nm) and mean ± standard deviation (SD) and ^∗^*p* < 0.05 according to the Kruskal–Wallis test followed by Dunn’s multiple comparisons test.

C57BL/6 mice were immunized with three inoculums of heat-killed *C. gattii* yeast with or without P3C4 (1 or 10 µg/mouse) at a two-week interval (on day 1, 14 and 28). The animals were infected with live *C. gattii* on day 42, as shown in Fig. 1A. Serum was used to measure the levels of IgM (Fig. 1B) and IgG (Fig. 1C) anti-GXM at 14 days post-immunization (day 35), and the levels detected in mice immunized and treated with adjuvant P3C4 did not differ from those of mice immunized alone (Figs. 1B–1C). In addition, serum was obtained 14 days after infection (day 56) to measure the levels of immunoglobulins IgG1, IgG2a, IgG2b, IgG3, IgA, IgM, and kappa/lambda chains. The levels of IgG1, IgG2a, and IgA isotypes in the mice that received 1 µg of adjuvant P3C4 were significantly lower than those in the immunized group (Fig. 1D), whereas the levels of IgG2b, IgG3, IgM, and kappa and lambda isotypes did not change significantly between groups. Mice treated with 10 µg of adjuvant P3C4 showed increased levels of the kappa chain compared with the immunized group (Fig. 1D). Taken together, these data demonstrate that the administration of adjuvant P3C4 did not improve the serum levels of immunoglobulin isotypes with effector functions against fungi, and the levels of GXM-specific antibodies did not change.

### High doses of adjuvant P3C4 downregulate the levels of pro-inflammatory cytokines in the lungs

**Figure 2:**
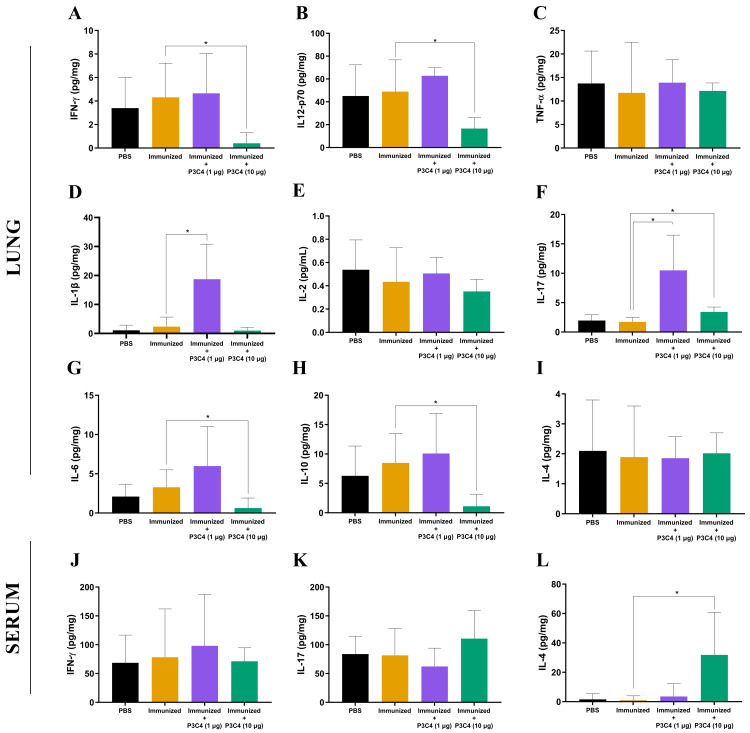
Cytokine levels in the lungs and serum of immunized mice in association with adjuvant P3C4. Mice were immunized with PBS or 2 × 10^7^ H.K.-*C. gattii* with or without Pam3CSk4 (1 µg or 10 µg) and infected with 1 × 10^5^ *C. gattii* yeast cells. Fourteen days post-infection, the lung homogenate supernatant was used to measure levels of (A) IFN- *γ*, (B) IL-12p70, (C) TNF-*α*, (D) IL-1*β*, (E) IL-2, (F) IL-17, (G) IL-6, (H) IL-10, and (I) IL-4, while the serum was used to measure (J) IFN-*γ* , (K) IL-17, and (L) IL-4 levels by ELISA. The concentration of the cytokines was calculated in relation to the organ mass expressed as pg/mg. The values were expressed as mean ± SD and ^∗^*p* < 0.05 according to the Kruskal–Wallis test followed by Dunn’s multiple comparisons test.

To evaluate the immunomodulation triggered by the immunization protocol, cytokine levels in the lungs and serum were quantified 14 days after *C. gattii* infection (day 56). The supernatant of the mouse lung homogenate was used to quantify the levels of IFN-*γ*, IL-12p70, TNF-*α*, IL-1*β*, IL-2, IL-17, IL-6, IL-10, and IL-4 (Figs. 2A–2I), while IFN-*γ*, IL-17, and IL-4 (Figs. 2J–2L) levels were measured in the serum. Immunization with heat-killed *C. gattii* yeast in association with 1 µg of P3C4 did not significantly change the levels of IFN-*γ*, IL-12p70, TNF-*α*, IL-2, IL-6, IL-10, and IL-4 (Fig. 2) in the lung tissue compared to the PBS and immunized groups. However, the adjuvant P3C4 −1 µg group had significantly higher levels of IL-1*β* (Fig. 2D) and IL-17 (Fig. 2F) in the lung tissue than the PBS and immunized groups. In contrast, administration of 10 µg of P3C4 as an adjuvant resulted in the reduction of IFN-*γ* (Fig. 2A), IL-12p70 (Fig. 2B), IL-6 (Fig. 2G), and IL-10 (Fig. 2H) levels in the lungs. Interestingly, the levels of IL-17 detected in the lung tissue of mice that received 10 µg of adjuvant P3C4 remained elevated in comparison to those in the immunized group (Fig. 2F), which was also observed in the P3C4-1 µg group. The downregulation of pro-inflammatory cytokines in the lungs promoted by the administration of 10 µg of P3C4 favored an increase in the levels of IL-4 in the serum, compared to the immunized group (Fig. 2L). Thus, the administration of 1 µg of P3C4 as an adjuvant contributed to the predominance of Th17-associated cytokines in the lung tissue, and pro-inflammatory cytokines essential to combat cryptococcosis were negatively regulated in lung tissue in the P3C4 −10 µg group.

### The frequency of TLR2+ cells in the lungs is altered by administration of the adjuvant P3C4

**Figure 3:**
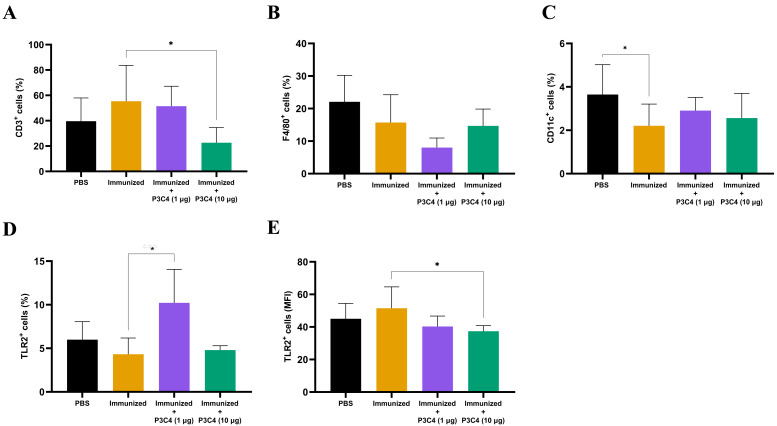
Frequency of positive cells for CD3, F4/80, CD11c or TLR2 in the lungs of immunized mice in association with adjuvant P3C4. C57BL/6 mice were treated with PBS or immunized with H.K.-*C. gattii* yeasts with or without association of either 1 *μ*g or 10 *μ*g of Pam3CSk4. After 14 days of infection with *C. gattii* (day 56), the lungs were collected and used for the preparation of a cell suspension to phenotype pulmonary leukocytes through flow cytometry. Cell suspensions were incubated with the following monoclonal antibodies: (A) anti-CD3 (PE-Cy5 Rat anti-Mouse CD3), (B) anti-F4/80 (FITC Rat anti-Mouse F4/80), (C) anti-CD11c (PE Hamster Anti-Mouse CD11c) and (D–E) anti-TLR2 (PE Rat Anti-Mouse TLR2). The frequency of positive cells was expressed as percentage (%) and the mean fluorescence intensity as MFI. The values are expressed as mean ± SD and ^∗^*p* < 0.05 according to the Kruskal–Wallis test followed by Dunn’s multiple comparisons test.

Since alterations in cytokine levels were observed in the lungs of immunized mice that received adjuvant P3C4, the frequency of alveolar macrophages, T cells, and TLR2+ cells could indicate immunomodulatory activity induced by 1 and 10 µg of P3C4. Therefore, a suspension of pulmonary leukocytes obtained from each group was incubated with anti-CD3, anti-F4/80 or anti-CD11c antibodies, and the frequency of T cells, alveolar macrophages and dendritic cells was measured by flow cytometry (Figs. 3A–3C). On day 56, the frequency of T cells (Fig. 3A) was decreased in the P3C4-10 µg group. The frequency of alveolar macrophages (Fig. 3B) and dendritic cells (Fig. 3C) measured in the P3C4 −1 µg and P3C4 −10 µg groups was not significantly different from that in the immunized and PBS groups. In contrast, immunized mice that received 1 µg of P3C4 had a significant increase in the frequency of TLR2^+^ cells compared with the immunized group, but not in the P3C4 −10 µg group (Fig. 3D). The level of TLR2 expression was determined by mean fluorescence intensity (MFI), and the immunized mice that received 10 µg of adjuvant P3C4 had a lower expression of TLR2 within pulmonary leukocytes than the immunized group (Fig. 3E). These data demonstrated that adjuvant P3C4 modulates the frequency of TLR2^+^ cells in the lungs and 10 µg of adjuvant P3C4 decreases the frequency of T cells, whereas the phenotypes of T cells (P3C4-1 *μ*g), alveolar macrophages and dendritic cells are unaltered.

### Low doses of the adjuvant P3C4 provide a predominance of the transcription factor Foxp3 in the lung tissue

**Figure 4:**
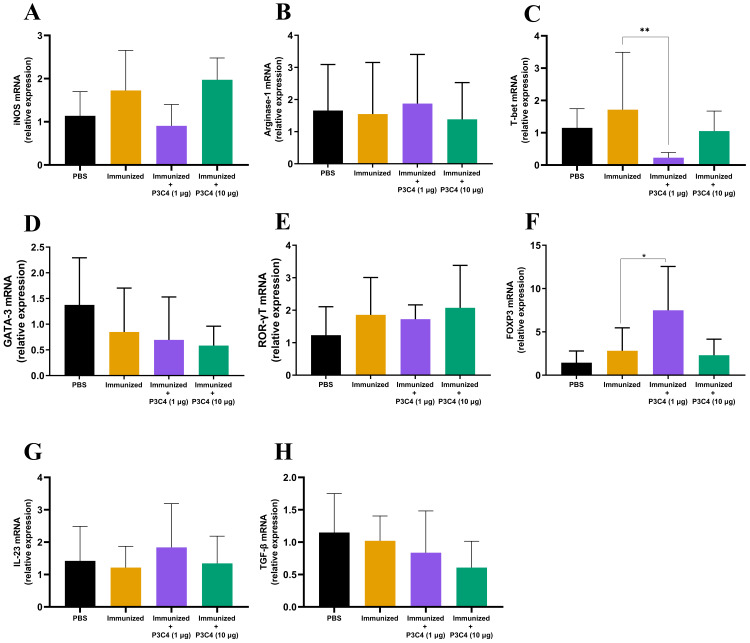
Effect of different doses of the adjuvant P3C4 in the relative expression of the differentiation markers of T cells and macrophages in the lung tissue. C57BL/6 mice were treated with PBS or immunized with H.K.-*C. gattii* yeasts with or without association of either 1 *μ*g or 10 *μ*g of Pam3CSk4. After 14 days of infection with *C. gattii* (day 56), the lungs were collected for RNA extraction using TRIzol™ reagent, followed by reverse-transcription into cDNA. The relative expression of iNOS (A), Arginase-1 (B), T-bet (C), GATA3 (D), ROR *γ* t (E), FOXP3 (F), IL-23 (G), and TGF-*β* (H) was determined by qRT-PCR. The ΔΔ Ct values were used to determine the relative expression, and *β*-actin transcript was used as endogenous control. The values are expressed as mean ± SD and ^∗^*p* < 0.05 according to the Kruskal–Wallis test followed by Dunn’s multiple comparisons test.

In addition to evaluating the phenotype of T cells and alveolar macrophages in the lung tissue, the differentiation of macrophages and CD4^+^ T cells was investigated by qRT-PCR using molecular markers that characterize M1/M2 subsets (iNOS and Arginase-1, respectively) and Th1/Th2/Th17/Treg profiles (T-bet, Gata-3, ROR-gt, and Foxp3, respectively). The relative expression of iNOS and Arginase-1 quantified in all groups did not differ (Figs. 4A–4B). Markers of CD4^+^ T cell differentiation in pulmonary tissue demonstrated that immunized mice that received 1 µg of adjuvant P3C4 showed a reduction in the relative expression of T-bet (Fig. 4C) and an increase in Foxp3 (Fig. 4F), which are related to Th1 and Treg cells, respectively. The relative expression of the markers of CD4^+^ T cell differentiation was not affected in the P3C4 −10 µg group compared with the control groups (Figs. 4C–4F). Additionally, the relative expression of IL-23 and TGF- *β*, which are involved in the initiation/maintenance of IL-17 production, was not significantly altered in either the P3C4 −1 µg or P3C4 −10 µg groups in comparison to the PBS and immunized groups (Figs. 4G–4H). Taken together, these data indicate that the administration of 1 µg of adjuvant P3C4 provided a predominance of transcription factors that characterize regulatory CD4+ T cells (Tregs) in the lung tissue.

### Adjuvant P3C4 is unable to contribute to the reduction of the *C. gattii* burden in the lungs

**Figure 5:**
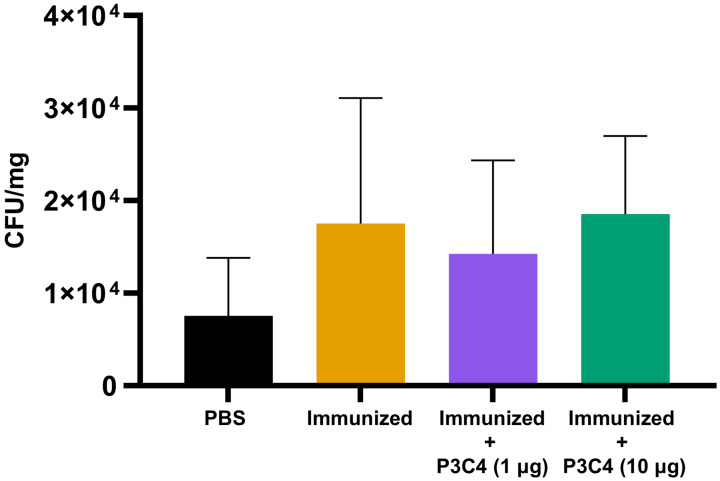
Fungal burden in the lungs of immunized mice in association with adjuvant P3C4 that were challenged with *C. gattii* infection. C57BL/6 mice were treated with PBS or immunized with H.K.-*C. gattii* yeasts with or without association of either 1 *μ*g or 10 *μ*g of Pam3CSk4. After 14 days of infection with *C. gattii* (day 56), the fungal burden in the lung homogenate was quantified by a colony forming unit (CFU) assay. The results were normalized in relation to the organ mass (CFU/mg). The values are expressed as mean ± SD and ^∗^*p* < 0.05 according to the Kruskal–Wallis test followed by Dunn’s multiple comparisons test.

Previous studies have demonstrated that the regulatory effect of Treg cells in the lung microenvironment of cryptococcosis can modulate allergic airway inflammation by suppressing Th2 cells and controlling *C. neoformans* burden (Schulze et al., 2014a; Schulze et al., 2014b; Schulze et al., 2016). Therefore, in the present study, adjuvant P3C4-1 µg induced an increase in the expression of the Foxp3 transcription factor in the lungs, which could indicate the differentiation of Treg cells; otherwise, the production of IL-17 and IL-1*β* was increased in the P3C4 −1 µg group. These factors influence the balance of the immune response against cryptococcosis, which requires quantification of the fungal burden in the lungs to evaluate the effect of adjuvant P3C4 in the control of *C. gattii* infection. The lung homogenates of immunized mice that received 1 µg or 10 µg of adjuvant P3C4 were obtained 14 days after *C. gattii* infection (day 56) to measure the fungal burden by the CFU assay. The groups with 1 µg or 10 µg of adjuvant P3C4 showed a pulmonary *C. gattii* burden that was not significantly altered when compared with the immunized group (Fig. 5). Therefore, the current findings related to the immunomodulatory activity of adjuvant P3C4 in the lungs show that it does not aid in the control of *C. gattii* infection.

### Low doses of the adjuvant P3C4 reduced the percentage of pulmonary parenchymal affected by inflammatory infiltrate

**Figure 6:**
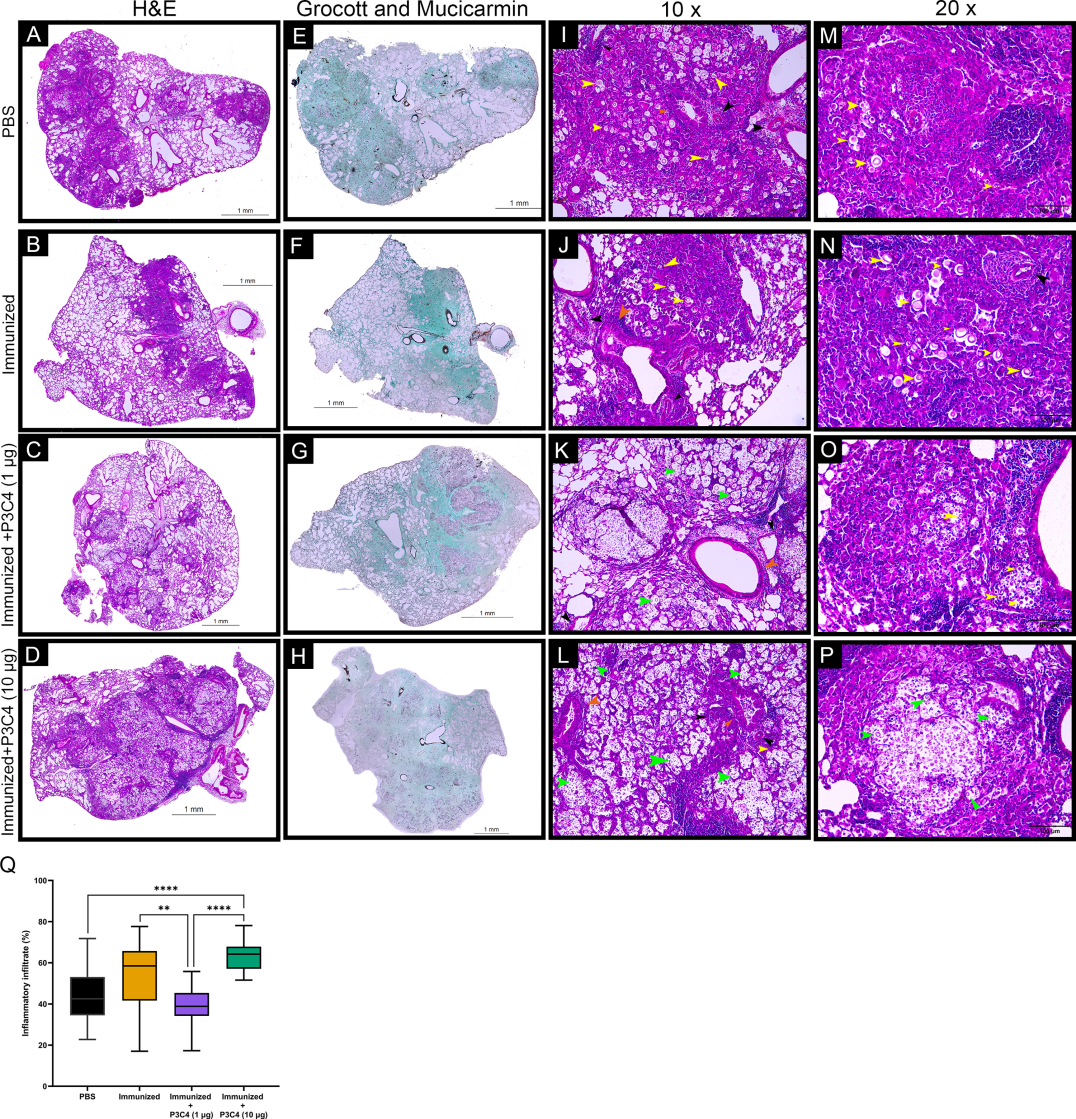
Histopathological and morphometric analysis of the lungs of immunized mice in association with adjuvant P3C4. Representative lung sections from PBS, Immunized, Immunized + P3C4 (1 µg), and Immunized + P3C4 (10 µg) groups. The total lung was acquired at 10x magnification, and the sections were stained with hematoxylin and eosin (H&E) (A–D) or stained with Grocott and Mucicarmine (E–H). The lung sections at 10x magnification (I–L) and 20x magnification (M–P) demonstrate the presence of *C. gattii* in the pulmonary parenchymal (yellow arrows) and alveolar spaces (green arrows). (I–P) Perivasculitis (black arrows) and peribronchiolitis (orange arrows) were observed in all groups studied, which can be representative as visualized in the panels I to P. (Q) Morphometric analysis of the lung tissue and the percentage of the pulmonary parenchymal was evaluated in a semi-automated quantitative way using ImageJ Fiji. Values were expressed as mean ± SD. ** *p* < 0.01, **** *p* < 0.0001, and non-significant (ns).

Considering that *C. gattii* has a predilection for the pulmonary tissue, histopathological and morphometric analysis of the lungs were performed to evaluate the architecture of the lungs and the distribution of inflammatory infiltrate throughout whole tissue. In all groups evaluated, the histopathological analysis demonstrated focal and multiple pulmonary nodular lesions in the sections (Figs. 6A–6D). PBS, Immunized, and Immunized + P3C4 −1 µg and −10 µg groups presented multiple nodular lesions with either low or high concentrations of inflammatory infiltrate composed, respectively, of mononuclear cells surrounded or robust lymphocytes infiltration with the presence of neutrophils (Figs. 6A–6D); moreover, the nodular lesions contained oval or rounded yeast cells diffusely distributed or restricted to the vacuolated cytoplasm of macrophages (Figs. 6I–6P). In addition, perivasculitis and peribronchiolitis were observed in all groups studied (Figs. 6I–6P). To visualize the distribution of fungi, the sections were stained with Grocott and Mucicarmine that demonstrated high frequency of *C. gattii* within the nodule lesions as observed in the Immunized + P3C4 −1 µg and −10 µg groups (Figs. 6E–6H). Specifically, the nodules lesions and the distribution of fungi were similar between PBS and untreated groups, whereas Immunized + P3C4 −1 µg and −10 µg groups had more prevalent focal nodular lesions with high frequency of *C. gattii* in the alveolar spaces (Figs. 6I–6P). These findings were complemented by the morphometric analysis of the percentage of inflammatory infiltrate throughout the whole tissue, and the mice that received 1 µg of adjuvant P3C4 showed a lower percentage of inflammatory infiltrate in relation to the area of pulmonary parenchymal, compared to Immunized and P3C4 −10 µg groups (Fig. 6Q). Taken together, these data indicate that different doses of adjuvant P3C4 can alter the distribution of inflammatory infiltrate in the lungs and the frequency of *C. gattii* yeast in the alveolar spaces.

## Discussion

The TLR2 agonist P3C4 can polarize macrophages to the M1 subset, characterized by the expression of high levels of iNOS, production of pro-inflammatory mediators, and activation of the NF- *κ*B pathway (da Silva et al., 2017; de Campos et al., 2020). P3C4 can also directly trigger the effector function of Th1 cells through the activation of MyD88 and MAPK, which promotes the production of remarkable levels of IL-2, IL-12, IFN-*γ*, and TNF-*α* (Imanishi et al., 2007). Moreover, Th17 cells express higher levels of TLR2/TLR1 and TLR2/TLR6 dimers than Th1 and Th2 cells, and TLR2 agonists intensify the expression of ROR *γ*t in Th17 cells, which increases IL-17 levels (Bird, 2010; Reynolds et al., 2010). The development and maintenance of Th1 and Th17 cells induced by TLR2 agonists should be evaluated as a mechanism to control cryptococcosis, although the immunomodulatory activity of TLR2 agonists in macrophages could be more relevant in combating cryptococcosis. Therefore, the capacity of TLR2 agonists to modulate the adaptive and innate immune responses should be investigated in the development of immunotherapeutic strategies against cryptococcosis, which can impact *Cryptococcus* spp. resistance in response to conventional antifungal drugs. The current study evaluated the administration of P3C4 as an adjuvant in an immunization protocol to treat *C. gattii* infection. Immunization was performed using heat-killed yeast of *C. gattii* associated with 1 µg or 10 µg of the adjuvant P3C4, which was administered intranasally with a two-week interval between each inoculum, and the animals were challenged with *C. gattii*. Immunization with 10 µg of adjuvant P3C4 resulted in low levels of IgG2a and IgM in the serum, and the levels of IFN-*γ*, IL-12p70, IL-6, and IL-10 in the lung tissue were reduced. Adjuvant P3C4 −10 µg did not alter the relative expression of transcription factors useful for inducing the differentiation of innate and adaptive immune cells in lung tissue. In contrast, adjuvant P3C4 −1 µg contributed to the predominance of Th17-associated cytokines in the lung tissue, and the transcription factor Foxp3 was also prevalent in the pulmonary tissue. Furthermore, a dose of 1 µg of P3C4 decreased the levels of IgG1, IgG2a, and IgA in mouse serum, and the levels of the transcription factor T-bet were also reduced in the lung tissue. In addition, the immunized mice that received 1 µg of P3C4 had a reduction in the percentage of inflammatory infiltrate in the pulmonary tissue. However, this lung tissue microenvironment is not sufficient to control *C. gattii* infection.

Vaccine strategies against several pathogens utilizing TLR2 agonists intranasally have been explored mainly in murine models, and it has been demonstrated that the stimulation of humoral and cellular immune responses could provide better protection to mice. These strategies could lead to high specific T cell proliferation and high levels of IFN-*γ*, IL-2, and IL-4 production (Cataldi et al., 2008; Cheng et al., 2011b), increased IL-17 levels and Th1/Th17 responses (Allen et al., 2018), and increased serum IgG and IgA levels (Cataldi et al., 2008). Intranasal administration of inactivated yeasts of *Cryptococcus* spp. was explored in a vaccination platform with mutated forms of yeast that improved the effector cellular immune response (Wang et al., 2019). In this context, intranasal administration is the most convenient route of choice for the present immunization protocol, as it is a safe route to induce a protective and immunogenic response against cryptococcosis.

The administration of synthetic TLR2 agonists can be relevant because of the induction of a pro-inflammatory immune response that is beneficial to combat cryptococcosis. Although soluble GXM, a major component of the *Cryptococcus spp.* capsule, can act as a ligand for TLR2, TLR4, and CD14 (Shoham et al., 2001), the role of TLR2 in the recognition of *Cryptococcus spp.* remains controversial. Schoffelen et al. (2013) revealed that clinical isolates of *C. gattii* induce human peripheral blood mononuclear cells (PBMCs) *in vitro* to produce high levels of cytokines related to Th1/Th17 responses, with a likely contribution of TLR4 and TLR9, but not TLR2. Yauch et al. (2004) demonstrated the importance of MyD88 during infection with *C. neoformans* with a minor role of CD14 and TLR2 . In contrast, Biondo et al. (2005) demonstrated an important role of TLR2 in the recognition of *C. neoformans*, and Fonseca et al. (2010) revealed that GXM from *C. gattii* induced a TLR2-mediated response and high production of NO by macrophages . Nevertheless, immunomodulation of TLR2 agonists can be a relevant strategy to assemble a robust inflammatory response, although a balance between pro- and anti-inflammatory responses is necessary. A previous study by our group revealed that TLR2 agonists can polarize peritoneal macrophages to the M1 subset, producing robust immunity; however, it did not result in a fungistatic effect on the growth of *C. gattii* (de Campos et al., 2020), and preliminary data from our group indicated that alveolar macrophages previously stimulated with TLR2 agonists did not reduce the growth of *C. gattii*. Alveolar macrophages represent more than 90% of the cells in the alveolar space (Nelson, Hawkins & Wozniak, 2020), and these cells could be the initial target of the vaccine proposed in the present study, suggesting that alveolar macrophages modulated by adjuvant P3C4 showed weak fungicidal activity.

Previous studies have shown that specific antibodies against GXM during infection can be produced at low titers; therefore, the administration of monoclonal antibodies specific to GXM has shown promising results in the control of cryptococcosis in murine models and clinical studies (Casadevall et al., 1998; Larsen et al., 2005; Shapiro et al., 2002). We evaluated serum levels of antibodies against GXM in the IgG and IgM subclasses. The animals in the immunized +P3C4 group had serum levels of anti-GXM IgG and IgM similar to those found in the control groups. Immunoglobulin isotyping was performed after mice were challenged with *C. gattii*, and the animals in the immunized +P3C4-1 µg group had reduced levels of IgG1, IgG2a, and IgA. The levels of these immunoglobulins indicate that immunization with P3C4-1 µg did not favor the production of effector isotypes against *C. gattii* infection, since IgG1 is the most abundant isotype after vaccination, and the IgG2a isotype is important for the recognition of carbohydrates.

We observed high levels of IL-17 and IL-1*β* in the lung tissue of animals that received 1 µg of adjuvant; however, the relative expression of the transcription factor ROR *γ*t did not change significantly among the groups. These data demonstrate that P3C4 induced the production of IL-17 in the lung tissue even in the absence of a predominance of the transcription factor ROR *γ*t that is a specific marker of Th17 cells. Previous studies have revealed that *C. neoformans* infection causes elevated levels of IL-17 in the lungs in the absence of Th17 cells (Wozniak et al., 2011), and increased levels of IL-17 and IL-1*β* were also detected in PBMC incubated with *C. gattii* (Schoffelen et al., 2013). Furthermore, vaccination with heat-killed *C. gattii* yeast containing a mutation in E3 ligase led to the induction of IL-17-producing CD4^+^ T cells that did not play an essential role in protecting mice against *C. gattii* infection (Wang et al., 2019).

The animals in the immunized +P3C4-1 µg group revealed a reduction in the relative expression of T-bet and increased relative expression of Foxp3, suggesting that immunization associated with P3C4-1 µg promotes the prevalence of regulatory T cells in the lung tissue along with a low frequency of Th1 cells, and this balance of differentiated CD4^+^ T cells does not contribute in combating *C. gattii* infection. These data are justified by other vaccination strategies that have been successful in controlling cryptococcosis through the induction of Th1 cells, along with high levels of IFN- *γ* (Wang et al., 2019; Wozniak et al., 2011). There is a possibility that regulatory T cells were induced in the lungs of the animals that received 1 µg of P3C4 due to an exacerbated pro-inflammatory immune response in the early stage of infection, thus leading to regulation of the immune response that suppresses Th1 cells. This hypothesis can be corroborated by the percentage of the inflammatory infiltrate in the lungs that was reduced in the immunized +P3C4-1 µg group indicating a lower recruitment of mononuclear cells to the pulmonary tissue after 14 days of *C. gattii* infection. In addition, the capacity of TLR2 activation to induce the production of IL-10 by APCs and T cells associated with the presence of pro-inflammatory cytokines can maintain the suppressive function of Treg cells, and this regulation can be modified as verified in the current work that showed a discrepancy in the balance of immune response between the adjuvant P3C4 at different concentrations. An interesting finding is the ability of Treg cells to suppress the Th2 response through the suppression of IRF-4 in the lungs, which promotes the growth of *C. neoformans* (Schulze et al., 2014a; Schulze et al., 2014b; Wiesner et al., 2016). However, the role of Tregs in the context of *C. gattii* infection progression must be well studied. In addition, evaluation of animals that received 10 µg of adjuvant P3C4 demonstrated an absence of high levels of relative expression of Foxp3, T-bet, and ROR *γ*t. Administration of 10 µg of P3C4, associated with immunization, maintained elevated levels of IL-17 in the lungs after *C. gattii* infection; however, the cytokine profile in the lungs was characterized by a reduction in the levels of IFN-*γ*, IL-12p70, IL-6, and IL-10. These findings suggest an immunosuppressive effect in the lungs through high concentrations of P3C4 associated with inactivated yeasts of *C. gattii*. Finally, the high IL-4 serum levels in the animals of the immunized group in association with 10 µg of P3C4 and infected with *C. gattii* reinforce the suppressive effect of the TLR2 agonist in the induction of cytokines and Th1 cells. Therefore, the immunomodulatory effect of adjuvant P3C4 is related to an immune response with suppressive/regulatory characteristics after *C. gattii* infection, which impairs the control of cryptococcosis.

## Conclusions

In this study, mice were immunized with heat-killed yeast of *C. gattii* associated with different doses of TLR2 agonist that should induce a predominant pro-inflammatory response, which is considered beneficial to combat cryptococcosis. We demonstrated that the immunomodulatory effect of adjuvant P3C4 occurs in the lungs of immunized mice, but it did not culminate in protection against *C. gattii* infection, and suppressive/regulatory characteristics in the microenvironment of pulmonary tissue were remarkable after *C. gattii* infection.

##  Supplemental Information

10.7717/peerj.14778/supp-1 Supplemental Information 1 Raw data

10.7717/peerj.14778/supp-2 Supplemental Information 2 Author Checklist

## Additional Information and Declarations

### Data Availability

The following information was supplied regarding data availability: The raw measurements are available in the Supplementary File.
